# Research hotspots and frontiers of vagus nerve stimulation in stroke: a bibliometric analysis

**DOI:** 10.3389/fnins.2024.1510658

**Published:** 2024-12-11

**Authors:** Mingyue Liu, Mengya Liu, Bohan Zhang, Mingzhu Fang, Ke Chen, Yishen Zhang, Qian Wang, Chunyan Tian, Liang Wu, Zhe Li

**Affiliations:** ^1^Department of Sports Rehabilitation, Beijing Xiaotangshan Hospital, Beijing, China; ^2^Department of Rehabilitation Medicine, The Fifth Affiliated Hospital of Zhengzhou University, Zhengzhou, China; ^3^School of Nursing, Centre for Smart Health, The Hong Kong Polytechnic University, Hong Kong SAR, China; ^4^Henan Rehabilitation Clinical Medicine Research Center, Zhengzhou, China

**Keywords:** vagus nerve stimulation, stroke, bibliometric analysis, hotspots and trends, CiteSpace

## Abstract

**Background:**

Vagus nerve stimulation (VNS) has emerged as a promising therapeutic approach for stroke treatment, drawing significant attention due to its potential benefits. However, despite this growing interest, a systematic bibliometric analysis of the research landscape is yet to be conducted.

**Methods:**

We performed a comprehensive search of the Web of Science Core Collection (WoSCC) database for literature published between January 1, 2005, and August 31, 2024. CiteSpace and the Bibliometrix package in R software were used to generate knowledge maps and conduct a bibliometric analysis. This analysis focused on publication output, geographic distribution, institutional involvement, author and co-cited author networks, journal and co-cited journal relationships, co-cited references, and keyword trends.

**Results:**

During the study period, 316 publications on VNS in stroke were identified, authored by 1,631 researchers from 1,124 institutions across 172 countries or regions. The number of publications showed steady growth, with the United States of America (USA) leading as the primary contributor. The University of Texas System emerged as the most active research institution. *Frontiers in Neuroscience* published the highest number of articles, while *Stroke* had the most citations. Professor Michael P. Kilgard authored the largest number of papers and was also the most frequently cited researcher. The main research trends focus on investigating VNS mechanisms via animal models and exploring its application in improving post-stroke sensorimotor function in the upper limbs. Moreover, VNS is showing promise in enhancing non-motor functions, such as swallowing, speech, and cognition, while addressing complications like post-stroke insomnia, depression, and disruptions in gut microbiota.

**Conclusion:**

This bibliometric study offers a comprehensive overview of the research landscape and emerging trends in VNS for stroke rehabilitation, providing a solid foundation and reference point for future research directions in this field.

## 1 Introduction

Stroke remains one of the leading causes of long-term disability globally, placing a substantial burden on healthcare systems (GBD 2019 Stroke Collaborators, 2021). While notable advancements have been made in acute stroke units and imaging diagnostics, the development of effective rehabilitation therapies for post-stroke recovery has not kept pace (Boulanger et al., 2018; Soun et al., 2021). Consequently, stroke rehabilitation has increasingly focused on the development of evidence-based strategies aimed at reducing stroke-induced functional impairments, enhancing patients’ ability to perform daily activities, and facilitating their participation in social activities (Stinear et al., 2020). In response to this demand, neuromodulation techniques, such as transcranial magnetic stimulation (TMS), transcranial direct current stimulation (tDCS), and vagus nerve stimulation (VNS), have gained significant traction in both basic and clinical neuroscience, offering novel therapeutic approaches for post-stroke rehabilitation (Begemann et al., 2020; Ahmed et al., 2023).

The vagus nerve, the most extensively distributed cranial nerve pair in the human body, innervates the largest number of effector organs. Approximately 80% of its fibers are afferent, with ascending pathways that relay in the nucleus of the solitary tract in the brainstem. These pathways project to the noradrenergic locus coeruleus system, thereby influencing the limbic system, thalamus, and extensive cortical networks (Carron et al., 2023). VNS refers to any method of stimulating the vagus nerve and is categorized into invasive Vagus Nerve Stimulation (iVNS) and transcutaneous Vagus Nerve Stimulation (tVNS). Moreover, tVNS can be further subdivided into transcutaneous cervical Vagus Nerve Stimulation (tcVNS) and transcutaneous auricular Vagus Nerve Stimulation (taVNS) (Butt et al., 2020). iVNS involves the application of electrical impulses to one side of the vagus nerve using specific parameters. Considering the potential impact on cardiac function, anatomical stability, and surgical safety, the left vagus nerve is typically chosen as the target (Hammer et al., 2015). tVNS, due to its non-invasive nature, can be performed bilaterally (Baig et al., 2023). VNS has emerged as a particularly promising strategy and has been approved by the U.S. Food and Drug Administration (FDA) for the treatment of conditions such as partial epilepsy, depression, and primary headaches (Johnson and Wilson, 2018; Fattorusso et al., 2021). Increasing clinical evidence suggests that VNS can enhance recovery from neural injuries, including stroke, thus reducing associated health risks and alleviating the medical burden (Keute and Gharabaghi, 2021). More specifically, VNS has been applied to improve motor function in stroke patients, facilitating better limb movement and coordination (Abdullahi et al., 2023; Ahmed et al., 2023). It has also shown potential in addressing cognitive impairments after stroke, such as memory deficits, attention problems, and executive dysfunction (Li Z.-D. et al., 2022). Additionally, VNS plays a critical role in emotional regulation, alleviating symptoms of depression and anxiety, which are commonly experienced by stroke survivors (Liu C. et al., 2024). Its application in pain management has also yielded favorable outcomes, reducing chronic pain often associated with stroke (Dodick, 2018). Moreover, VNS has proven effective in improving autonomic nervous system functions, including heart rate and blood pressure regulation (Farmer et al., 2021). In rehabilitation therapy, VNS is frequently used as an adjunct to traditional physical and occupational therapies, amplifying therapeutic outcomes. During the acute phase of stroke, VNS may help reduce the severity and extent of brain injury, and in the longer term, it supports sustained functional recovery.

Despite the increasing interest in the application of VNS in stroke therapy, comprehensive systematic and quantitative analyses in this area remain scarce. This gap limits our understanding of the current research landscape and the identification of future trends. Bibliometrics, which uses mathematical and statistical methods to analyze literature both quantitatively and qualitatively, can provide valuable insights into research trends and emerging areas by mapping citation networks (Donthu et al., 2021). Compared with traditional reviews, bibliometric analysis excels at revealing the internal structure and potential connections within extensive bodies of literature (Ellegaard and Wallin, 2015). Previous bibliometric studies have explored the use of innovative technologies such as repetitive transcranial magnetic stimulation, virtual reality, and robotics in stroke rehabilitation, as well as the clinical applications of VNS (Huang et al., 2016; Zheng et al., 2020; Li R. et al., 2022; Du et al., 2024; Wen et al., 2024). However, to date, no bibliometric analysis has specifically focused on the role of VNS in stroke therapy. To address this gap, the present study employs bibliometric methods, utilizing tools such as CiteSpace and R, to visually analyze the literature on VNS in stroke rehabilitation. By constructing knowledge maps and identifying research hotspots, this study aims to provide theoretical support and guide future innovations in this evolving field.

## 2 Materials and methods

### 2.1 Data sources and search strategy

**Figure 1:**
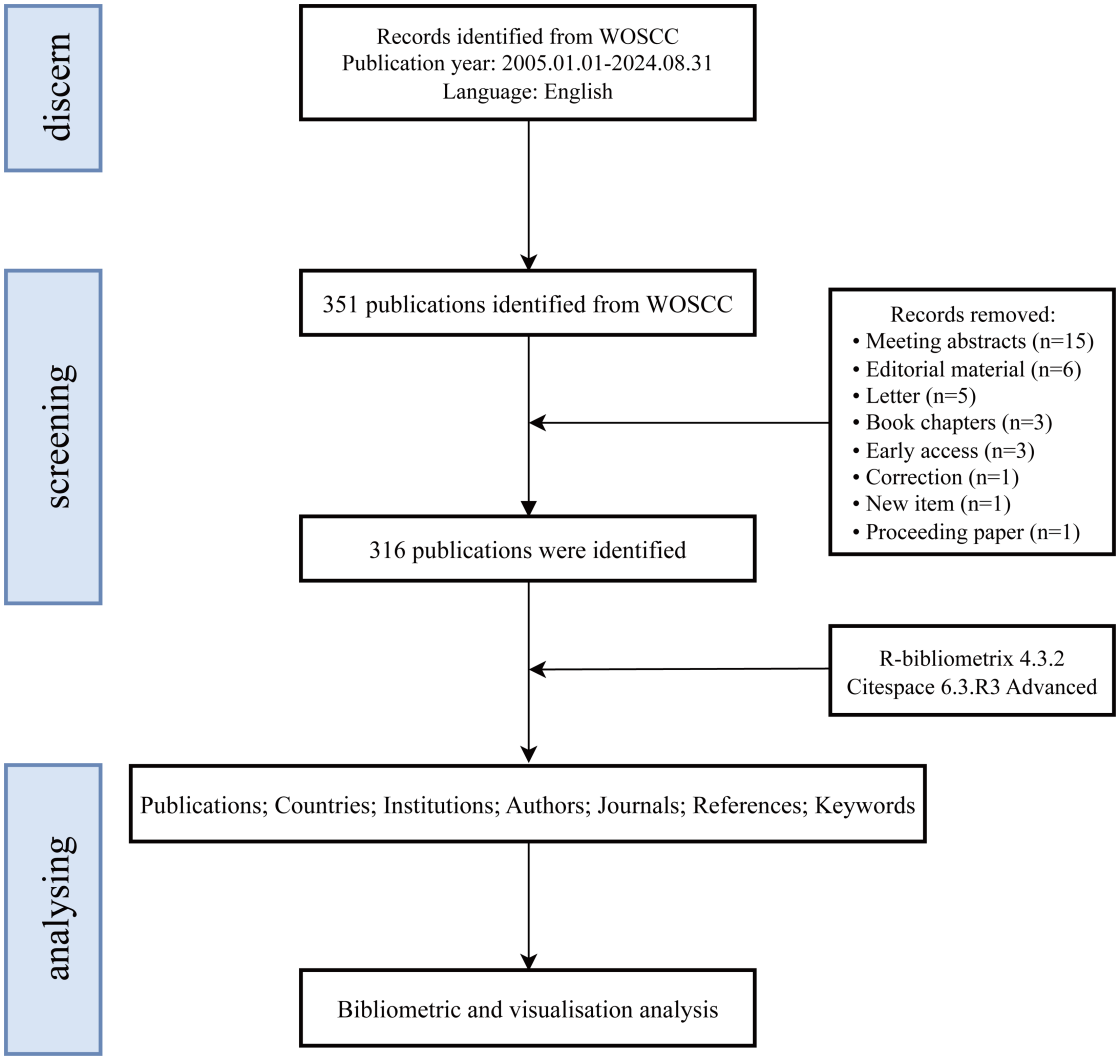
Flow diagram for the screening and analysis of publications.

Given that the Web of Science Core Collection (WoSCC) and Scopus are widely regarded as the leading bibliometric databases, the use of alternative databases that do not provide co-citation data significantly limits the scope and depth of bibliometric analyses (Echchakoui, 2020). While Scopus is a comprehensive resource, it includes a substantial number of articles without impact factors, which may introduce a degree of uncertainty regarding the reliability of analytical results (AlRyalat et al., 2019). To ensure a systematic analysis, this study utilized the WoSCC, which encompasses the following sub-databases: the Science Citation Index Expanded (SCI-EXPANDED), the Social Sciences Citation Index (SSCI), the Arts & Humanities Citation Index (A&HCI), the Conference Proceedings Citation Index—Science (CPCI-S), the Emerging Sources Citation Index (ESCI), the Current Chemical Reactions Index (CCR-EXPANDED), and the Index Chemicus (IC). A topic search was conducted on September 1, 2024, using the query: TS = (Vagus Nerve Stimulation OR Vagus Nerve Stimulation* OR Stimulation*, Vagus Nerve OR Nerve Stimulation*, Vagus OR Vagal Nerve Stimulation* OR Stimulation*, Vagal Nerve OR Nerve Stimulation*, Vagal OR Transcutaneous auricular vagus nerve stimulation* OR Transcutaneous vagus nerve stimulation* OR Noninvasive vagus nerve stimulation* OR Transcutaneous cervical vagus nerve stimulation*) AND TS = (Stroke* OR Cerebrovascular Accident* OR Cerebral Stroke* OR Cerebrovascular Apoplexy OR Brain Vascular Accident* OR Cerebrovascular Stroke* OR Apoplexy OR Acute Stroke* OR Acute Cerebrovascular Accident* OR Ischemic Stroke* OR Brain Stem Infarction OR Hemorrhagic Stroke* OR Intracerebral Hemorrhagic Stroke* OR Subarachnoid Hemorrhagic Stroke*). The search covered publications from January 1, 2005, to August 31, 2024. Only articles classified as “original research” or “review articles” and published in English were included (Ozturk et al., 2024). The analysis focused on full-text information and references, with search results saved in plain text format. After applying inclusion criteria, 316 eligible records were selected. Figure 1 provides an overview of the publication screening process.

### 2.2 Data analysis

The selected literature was imported into CiteSpace software (version 6.3.R3 Advanced) and the Bibliometrix package in R software (version 4.4.1) for bibliometric analysis. To ensure the integrity and reliability of the data, a preprocessing step was carried out, which included unifying synonyms, removing irrelevant terms, and standardizing author names and institutional affiliations (Linnenluecke et al., 2020). CiteSpace, known for its ability to map knowledge, excels at identifying citation clusters, recognizing key knowledge nodes, and analyzing the spread and development of research (Moral-Munoz et al., 2020). Simultaneously, the Bibliometrix package in R was employed for quantitative analysis, offering insights into geographic distribution, author collaborations, publication trends, core journal identification, and institutional contributions (Gaviria-Marin et al., 2019). By combining the strengths of both CiteSpace and Bibliometrix, this study delivers a comprehensive overview of the research landscape, highlighting key focus areas, prominent topics, and emerging trends in VNS for stroke rehabilitation. The study also investigates the developmental trajectory and evolution of research within this domain.

## 3 Results

### 3.1 Annual publication growth trend

**Figure 2:**
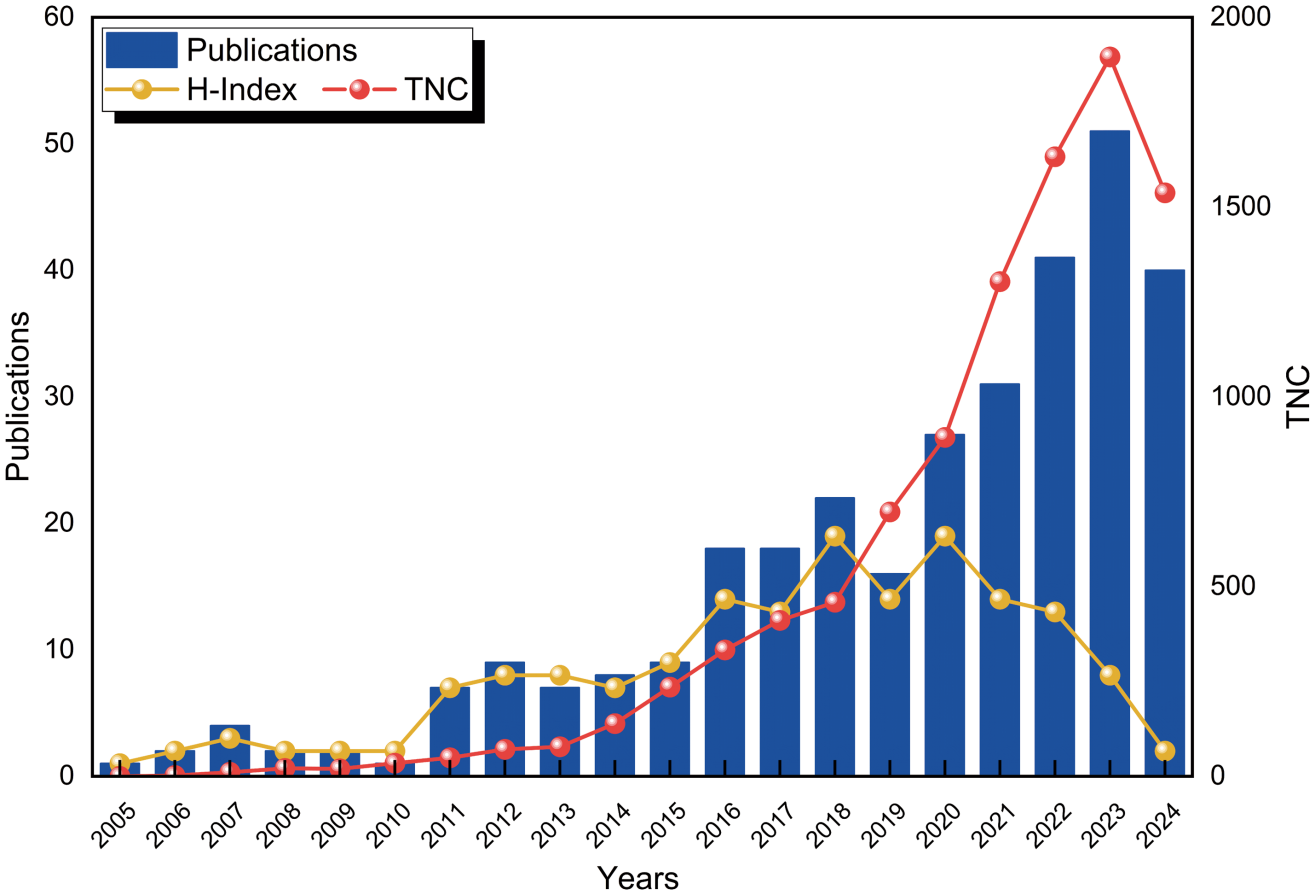
Trends in the number of publications and citation growth over time.

From 2005 to 2024, a total of 316 publications related to the application of VNS in stroke were identified in the WoSCC database. Figure 2 depicts the trends in the annual number of publications (Np), citations (Nc), and the Hirsch index (H-index). Between 2005 and 2010, the Np remained relatively low. However, a gradual increase was observed starting in 2011, with a minor decline in 2019, followed by a substantial surge post-2020, reaching a peak of 51 publications in 2023. The H-index exhibited a fluctuating upward trend from 2005 to 2015 and remained relatively stable between 2016 and 2022, reflecting consistent progress in research within this domain. A slight decrease in the H-index was noted after 2022, likely due to the limited timeframe. Similarly, the Nc gradually rose between 2015 and 2018, followed by a significant increase from 2018 onwards. These trends suggest that the application of VNS in stroke rehabilitation is garnering increasing scholarly attention and has the potential to drive future advancements in the field.

### 3.2 National or regional collaboration analysis

**Figure 3:**
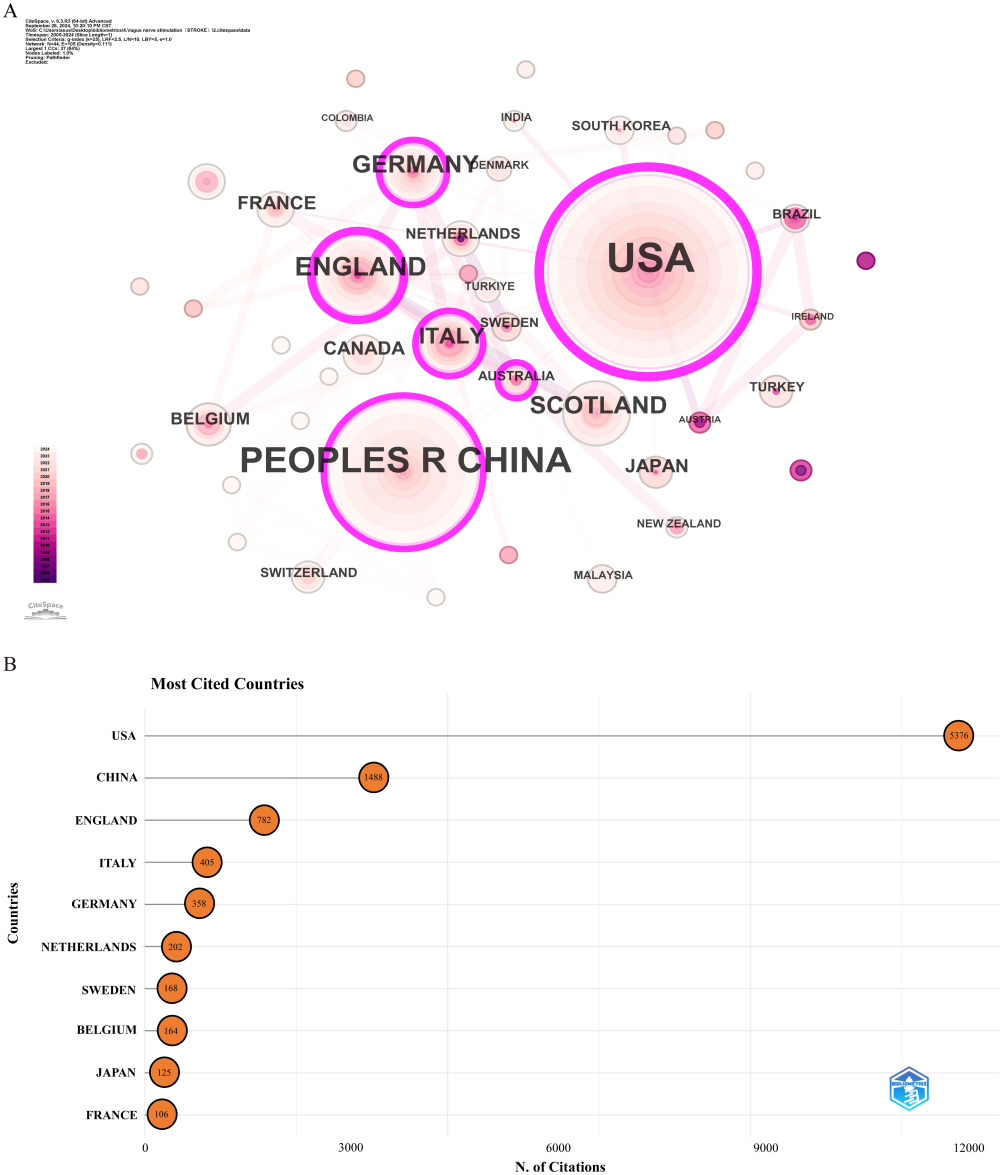
Analysis of countries involved in stroke-related VNS research. **(A)** Co-occurrence map of countries. The node size represents the frequency of co-occurrence, and connecting lines indicate co-occurrence relationships. **(B)** The top 10 countries ranked by citation count.

Over the past two decades, 172 countries or regions have engaged in research on the application of VNS in stroke. Table 1 lists the top 10 countries by Np and Nc. The USA (Np: 160) and China (Np: 86) ranked first and second, followed by England (Np: 40) and Germany (Np: 16). Figure 3A illustrates the international collaboration network, comprising 79 nodes and 444 links, where each node represents a country or region. The size of each node correlates with the volume of publications, and the links between nodes represent the degree of inter-country collaboration. Nodes with a purple outer ring signify high betweenness centrality (≥ 0.1), with the top five countries being the USA (0.69), England (0.33), China (0.17), Italy (0.12), and Germany (0.11). Combined with Figure 3B, it is evident that the USA leads in Np, Nc, and centrality, underscoring its prominent role in the field. European countries, including England, Germany, and Italy, have also demonstrated strong research capabilities. Notably, despite having a relatively lower Np, England’s high betweenness centrality highlights the quality and international influence of its research. Although China ranks second globally in terms of Np, its lower centrality may be attributed to its relatively recent entry into this field. In recent years, China’s increased investment in this area has driven rapid growth in Np, but the depth and breadth of its collaborative network still require further expansion.

**Table 1:** The top 10 countries producing the most publications on VNS in stroke research.

Rank	Countries(regions)	Np	Centrality	Nc	Countries (regions)
1	USA	160	0.69	5,376	USA
2	China	86	0.17	1,488	China
3	England	40	0.33	782	England
4	Germany	16	0.11	405	Italy
5	Italy	14	0.12	358	Germany
6	France	9	0	202	Netherlands
7	Japan	8	0	168	Sweden
8	Canada	8	0.03	164	Belgium
9	Belgium	6	0.02	125	Japan
10	Netherlands	5	0.01	106	France

### 3.3 Institutional collaboration analysis

**Figure 4:**
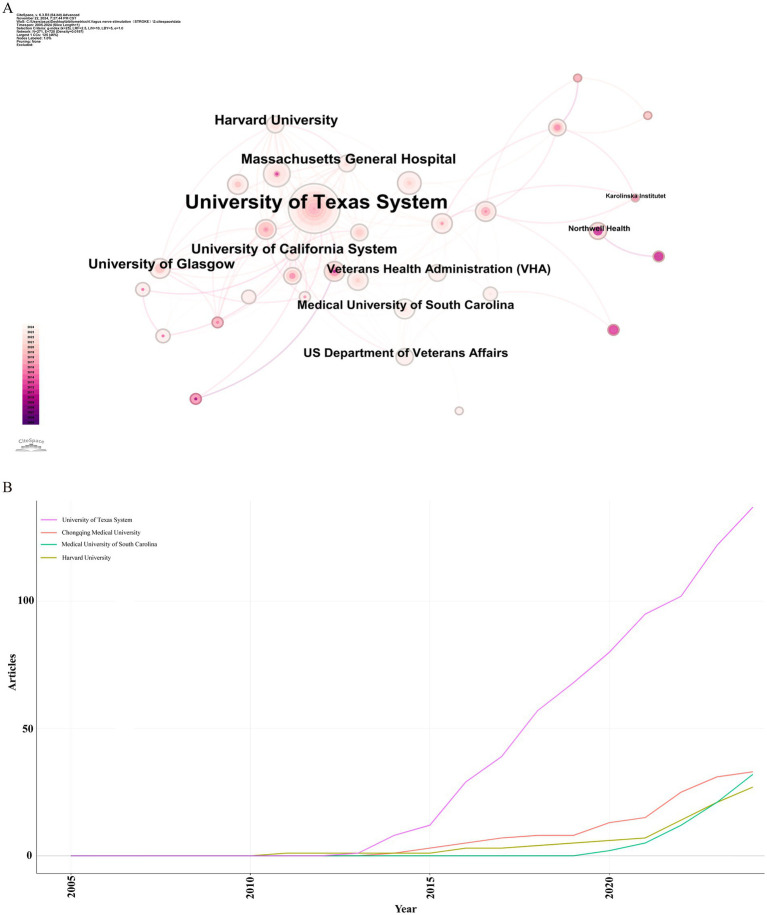
Institutional analysis of stroke-related VNS research. **(A)** Co-occurrence map of research institutions. **(B)** Temporal distribution of publications across various institutions.

Over the past two decades, 1,124 institutions published research on the application of VNS in stroke. Figure 4A presents the institutional collaboration network, primarily consisting of global higher education institutions. Table 2 highlights the top five institutions in terms of Np and Nc, primarily based in the USA, Europe, and China. Notably, the University of Texas System ranks first in both publication volume and citation count, demonstrating its significant academic influence in the field of VNS stroke research. Figure 4B illustrates the publication trends of these institutions, revealing a substantial increase in studies conducted by the University of Texas over the past 5 years, establishing it as the most collaboration-oriented institution, with research outputs that are poised to provide substantial contributions to the field in the future.

**Table 2:** The top 10 institutions producing the most publications on VNS in stroke research.

Rank	Institutions	Countries (regions)	Np	Centrality
1	University of Texas System	USA	57	0.01
2	Chongqing Medical University	China	22	0.01
3	Harvard University	USA	15	0.04
4	Massachusetts General Hospital	USA	14	0.02
5	University of California System	USA	13	0.05
6	University of Glasgow	England	13	0.04
7	Medical University of South Carolina	USA	12	0.04
8	US Department of Veterans Affairs	USA	12	0.03
9	Chinese Academy of Sciences	China	11	0
10	Veterans Health Administration (VHA)	USA	57	0.01

### 3.4 Author collaboration analysis

**Figure 5:**
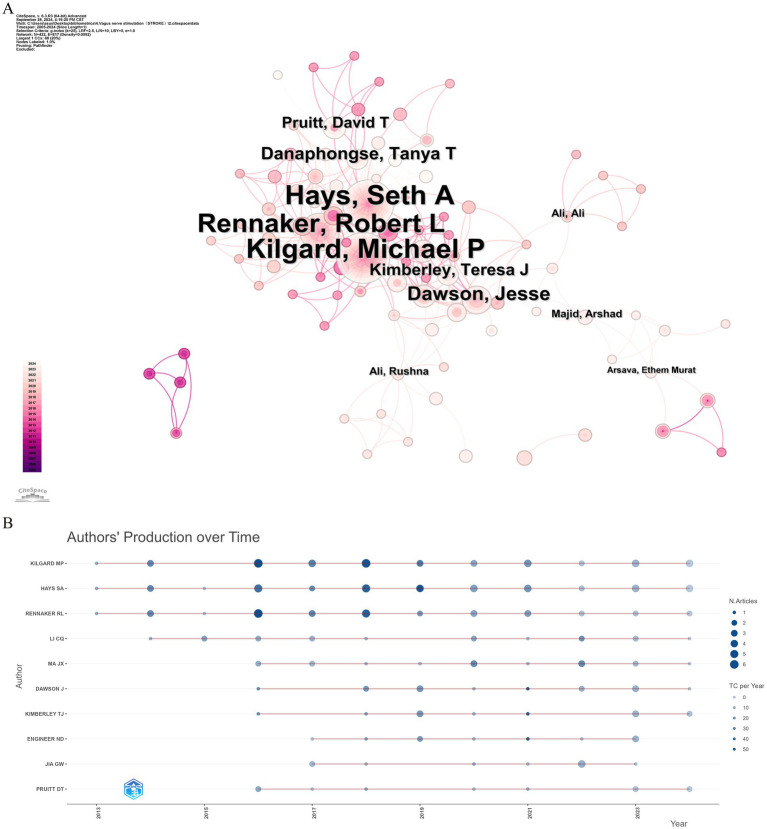
Analysis of author contributions in stroke-related VNS research. **(A)** Co-occurrence map of authors. **(B)** Examination of highly cited authors.

Over the past two decades, 1,631 scholars have participated in research related to VNS in stroke. Figure 5A displays the author collaboration network, while Table 3 lists the top ten authors by Np and Nc. Professor Michael P. Kilgard from the University of Texas leads with 38 publications and 916 citations, followed closely by Professor Seth A. Hays from the same institution (Np: 37, Nc: 861). These metrics highlight their substantial contributions to the field and underscore the widespread recognition of their research within the academic community. Figure 5B illustrates the annual publication trends of highly cited authors, indicating that Michael P. Kilgard, Seth A. Hays, and Robert L. Rennaker were among the early contributors to this domain and have maintained high research productivity, further attesting to the academic significance of their work.

**Table 3:** The top 10 authors with the most publications on VNS in stroke research.

Rank	Authors	Np	Centrality	Nc	Authors
1	Kilgard, Michael P	38	0.03	916	Kilgard MP
2	Hays, Seth A	37	0.02	861	Rennaker RL
3	Rennaker, Robert L	30	0.01	859	Hays SA
4	Dawson, Jesse	11	0.03	367	Khodaparast N
5	Li, Changqing	11	0	357	Hulsey DR
6	Ma, Jingxi	9	0	320	Dawson J
7	Danaphongse, Tanya T	8	0	299	Ruiz A
8	Badran, Bashar W	7	0	276	Kimberley TJ
9	Kimberley, Teresa J	7	0	259	Sloan AM
10	Engineer, Navzer D	6	0	230	Pierce D

### 3.5 Journal analysis

**Figure 6:**
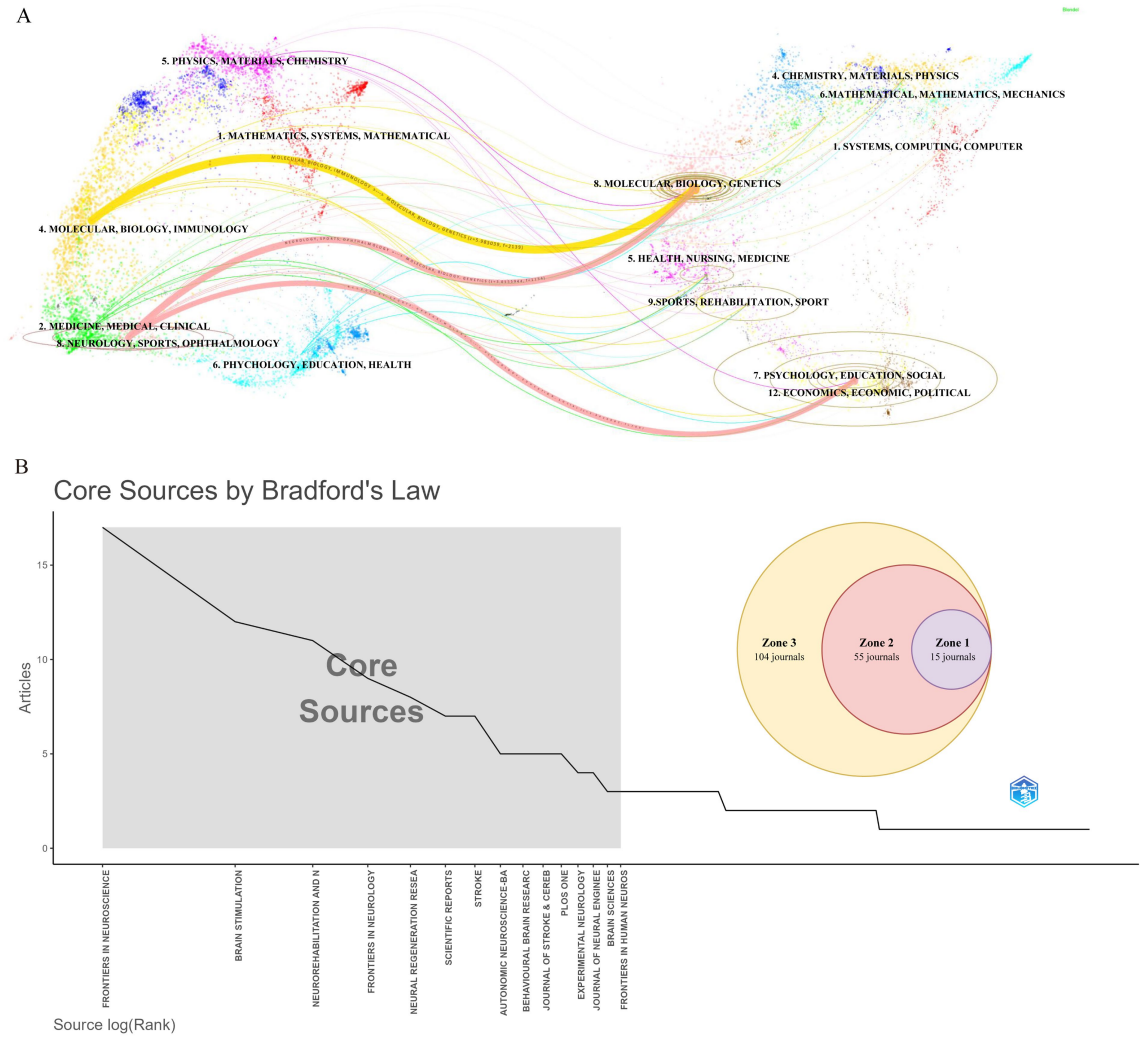
Journal-level analysis in stroke-related VNS research. **(A)** Dual-map overlay of journals. **(B)** Classification of journals according to Bradford’s law.

During the study period, publications related to VNS in stroke were distributed across 174 academic journals. Table 4 reveals that the journal with the highest number of publications is *Frontiers in Neuroscience* (Np: 17), followed by *Brain Stimulation* (Np: 12). Among the top 10 journals by citation count, seven have received more than 300 citations, with *Stroke* having the highest Nc (1,142). Figure 6A presents a dual-map overlay of these journals, offering a visual representation of their distribution, citation pathways, and thematic evolution (Chen and Leydesdorff, 2014). Generally, journals categorized under Neurology, Sports, and Ophthalmology frequently cite articles from Molecular Biology, Genetics, and Psychology, Education, and Social Sciences. Figure 6B categorizes journals into three zones based on Bradford’s Law, with Zone 1 encompassing 15 core journals, Zone 2 covering 55 secondary journals, and Zone 3 comprising 104 peripheral journals.

**Table 4:** The top 10 journals publishing the most papers on VNS in stroke research.

Rank	Journals	Np	Country	Nc	Journals
1	Front. Neurosci.	17	Switzerland	1,142	Stroke
2	Brain Stimul.	12	USA	687	Brain Stimul
3	Neurorehabil. Neural Repair	11	USA	418	Neurorehabil Neural
4	Front. Neurol.	9	Switzerland	376	Neurology
5	Neural Regen. Res.	8	China	364	Brain Res
6	Sci. Rep.	7	England	313	J Neurosci
7	Stroke	7	USA	310	Epilepsia
8	Auton. Neurosci. Basic Clin.	5	Netherlands	281	Nature
9	Behav. Brain Res.	5	Netherlands	275	PLOS ONE
10	J. Stroke Cerebrovasc. Dis.	5	USA	265	Lancet

### 3.6 Reference analysis

**Figure 7:**
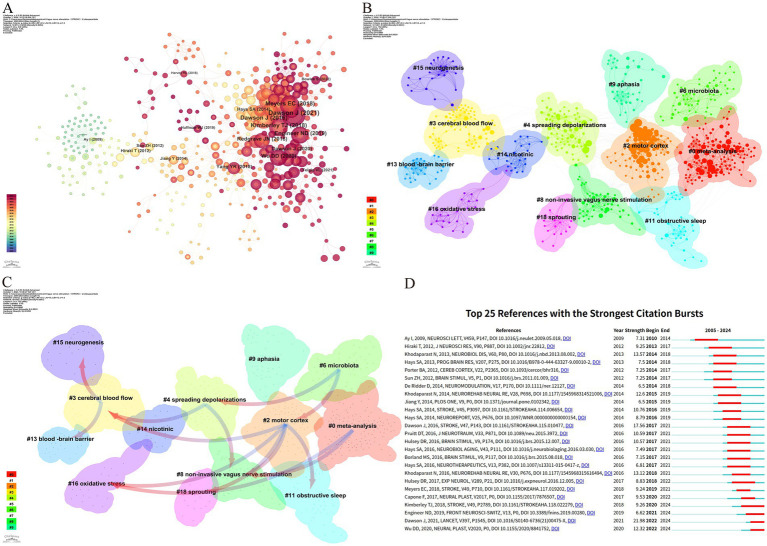
Reference analysis of VNS research in stroke. **(A)** Document co-occurrence network; **(B)** Clustering analysis based on document similarity, identifying 13 major clusters: #0 meta-analysis, #2 motor cortex, #3 cerebral blood flow, #4 spreading depolarizations, #6 microbiota, #8 non-invasive vagus nerve stimulation, #9 aphasia, #11 obstructive sleep, #13 blood–brain barrier, #14 nicotinic, #15 neurogenesis, #16 oxidative stress, and #18 sprouting; **(C)** Dependency analysis of clusters, showing that cluster #2 evolved from clusters #3, #8, #11, #16, and #18; **(D)** The top 25 most frequently cited documents, showing citation bursts with red bars indicating periods of heightened citation activity.

Figure 7A illustrates that the research predominantly focuses on the fundamental mechanisms of VNS and its potential application in enhancing motor function, demonstrating high intermediary centrality in this field. Co-citation cluster analysis provides a nuanced depiction of the knowledge structure within this research domain (Onan, 2019). Figure 7B categorizes the application of VNS in stroke research into 13 distinct clusters based on the relationships among the cited literature. The Q value (cluster module value) of 0.9066 indicates a significant clustering structure within this field. Additionally, the S value (mean profile value) of 0.9533 reflects a high degree of homogeneity among cluster members (Sabe et al., 2022). Together, the Q and S values form the foundation of the clustering analysis. The largest cluster, #0 meta-analysis, primarily comprises evidence-based medical studies on VNS for post-stroke motor recovery. Early clusters, such as #15 neurogenesis, #13 blood–brain barrier, #3 cerebral blood flow, and #16 oxidative stress, have progressively expanded into more closely related fields, including #4 spreading depolarizations, #8 non-invasive vagus nerve stimulation, and #18 sprouting. In recent years, the interconnections among research areas have become increasingly tight, with #0 meta-analysis, #2 motor cortex, #9 aphasia, and #11 obstructive sleep emerging as more centralized clusters.

Figure 7C, through the dependency analysis of co-citation clusters, clearly identifies current research hotspots and the evolutionary relationships among these clusters. Foundational studies such as #13 blood–brain barrier, #3 cerebral blood flow, #15 neurogenesis, and #16 oxidative stress form the early basis of this field and have gradually evolved into other clusters. The high linkage density of #2 motor cortex and #8 non-invasive vagus nerve stimulation, each evolving from multiple clusters and further developing into new clusters, suggests that they might be recent focal points in VNS stroke research. Clusters like #0 meta-analysis, #6 microbiota, and #9 aphasia have mainly evolved from other clusters but have not further branched out, potentially representing the frontier research in VNS for stroke.

Citation bursts refer to a phenomenon where the citation count of a particular paper significantly and suddenly increases over a specific period (Ozturk et al., 2024). Figure 7D lists the top 25 papers with the strongest citation bursts. The earliest citation burst appeared in 2010, with the strongest burst (strength = 21.98) observed in the research by Dawson J et al., published in 2021 in *The Lancet*, titled “Vagus nerve stimulation paired with rehabilitation for upper limb function after stroke: A randomized, triple-blind, sham-controlled trial.” This study confirmed the safety and efficacy of VNS in the recovery of upper limb function after ischemic stroke. Similarly, the study by Kimberley TJ et al., published in *Stroke* in 2018, titled “Vagus Nerve Stimulation for Arm Function Recovery After Ischemic Stroke: A Pivotal Randomized, Controlled Trial, “also exhibited a high citation burst (strength = 9.26) and has continually garnered attention since 2018. According to the results, 2016 saw the most citation bursts, followed by 2014 and 2021, indicating that high-burst articles from these years sparked significant research interest. Currently, three papers remain in a state of citation burst.

### 3.7 Keyword analysis

**Figure 8:**
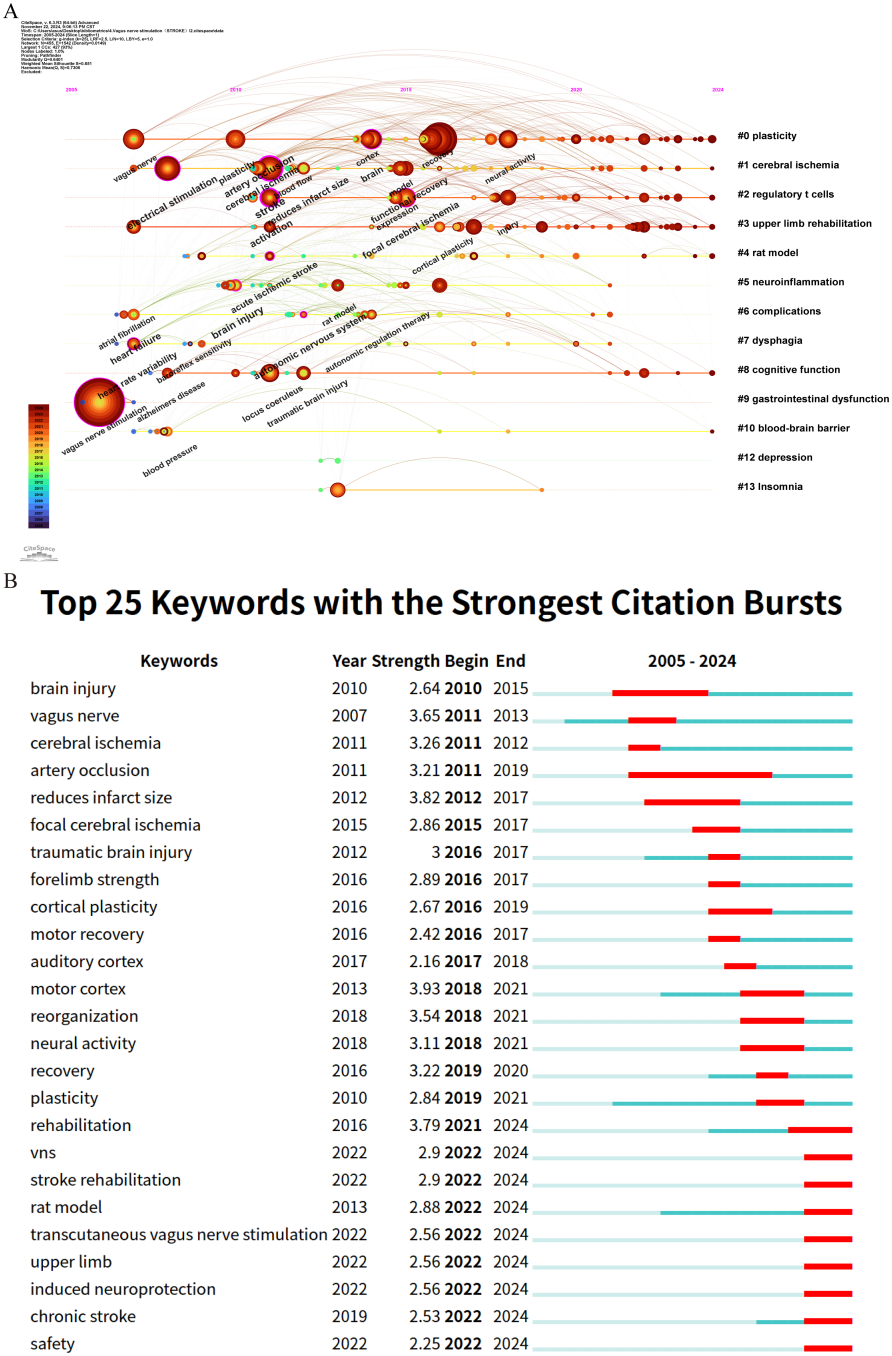
Keyword analysis in stroke-related VNS research. **(A)** Temporal evolution of keywords; **(B)** The top 25 keywords with the strongest citation bursts.

The timeline visualization of keywords is instrumental in examining their evolution and dynamics across various clusters (Jin et al., 2023). As depicted in Figure 8A, the progressive development of keywords related to VNS in the field of stroke research, along with the research foci at different stages, is clearly observable. Among the 14 identified clusters, 9 are still in an active research state, indicating that VNS remains a highly dynamic area within stroke research. The largest cluster, #0 plasticity, features early keywords such as “upper extremity,” “rehabilitation,” and “pre-motor and supplementary motor cortex,” signifying the initial research emphasis on motor function restoration. Cluster #4 rat model, the earliest investigated and the most explosively cited cluster, continues to be a primary research focus over the past 3 years. The newest cluster, #8 cognitive function, is also currently in a highly active phase. The evolution of research keywords reflects the trajectory of VNS studies in stroke research. Initially, the focus was predominantly on elucidating the neuropathological mechanisms underlying VNS in stroke. However, there has been a shift towards investigating its diversified clinical applications and evidence-based outcomes.

Keyword citation bursts, serving as a crucial indicator for identifying research hotspots and emerging trends, are depicted through red segments on the blue timeline, which visually represent the onset, duration, and cessation of these bursts (Wu et al., 2021). As shown in Figure 8B, among the top 25 keywords with the strongest citation bursts, “motor cortex” exhibits the highest burst strength (3.93), followed by “reduces infarct size” (3.82) and “rehabilitation” (3.79). Notably, keywords such as “rat model,” “transcutaneous vagus nerve stimulation,” “upper limb,” “induced neuroprotection,” and “safety” remain in an ongoing burst state, highlighting their sustained relevance and continued research interest.

## 4 Discussion

### 4.1 General information

This study represents the first bibliometric analysis of research on the application of VNS in stroke over the past two decades. From 2005 to 2010, the field was in its infancy, with a relatively limited research foundation. However, between 2011 and 2021, the annual publication volume on VNS in stroke research exhibited a fluctuating growth trend. Despite a temporary decline in 2019, the citation frequency of the literature continued to rise steadily, and the H-index remained consistently high, indicating that the field is gradually entering a systematic research phase. Since 2021, the number of relevant publications has surged, peaking at 51 papers in 2023 approximately seven times the number in 2013. These findings suggest that in recent years, the application of VNS in stroke has garnered increasing attention from researchers worldwide and is now in a phase of steady development.

A visual analysis at the national (or regional) level reveals a co-occurrence density of 0.11, highlighting significant global collaboration in VNS stroke research, which bodes well for the long-term development of the field. The analysis further indicates that the USA, China, and England are the leading countries in VNS stroke research. The top 10 contributing countries include six European nations, two from the Asia-Pacific region, and two from the Americas. Countries with high centrality play a pivotal role in the global collaborative network, with the USA being the most active, driven by the prominence of its research institutions. Among the top 10 global research institutions, seven are from the USA. Although China ranks second globally in terms of publication volume, its intermediary centrality remains relatively low, likely due to China’s substantial investments in the field in recent years. Despite the rapid increase in Chinese publications, international academic collaboration remains relatively limited. Moreover, among the top 10 research institutions globally, seven are from the USA, two from China, and one from England. The University of Texas System occupies a dominant position in global VNS stroke research collaboration. The inter-institutional co-occurrence graph reveals that collaborative patterns among institutions are largely concentrated within individual countries. Therefore, there is a need for research institutions from various countries to strengthen cross-national collaboration and communication to jointly advance the application of VNS in stroke treatment.

From the perspective of authorship, Professors Michael P. Kilgard, Seth A. Hays, and Robert L. Rennaker from the University of Texas have made significant contributions to the field of VNS stroke research, with high Nc and Np, cementing their pioneering status in the field. Their preclinical and clinical studies on VNS combined with rehabilitative training have demonstrated that VNS significantly enhances motor skill recovery and neural circuit remodeling, especially when integrated with rehabilitation (Hays et al., 2014; Kilgard et al., 2018; Morrison et al., 2019). Their research has elucidated the positive effects of VNS on neuronal circuits, promoting its clinical application as an innovative stroke treatment. The co-occurrence graph of core authors reveals distinct research groups led by core researchers. While scholars are active within their respective teams, cross-group collaboration needs to be further strengthened to foster broader development and innovation in the field. In this context, widespread collaboration among research institutions is particularly crucial. Such institutional partnerships can mitigate the escalating costs associated with research infrastructure while fostering cooperation across specialized fields, including basic and clinical medicine. Moreover, these collaborations can act as conduits, facilitating interactions among researchers and establishing the groundwork for new joint ventures in diverse research areas. By promoting collaboration, these institutional partnerships and joint projects enable scientists and scholars to navigate various research systems, institutions, and funding opportunities, thereby enhancing overall research capacity.

Journal analysis shows that *Frontiers in Neuroscience* has published the most papers on VNS in stroke research, followed by *Brain Stimulation* and *NeuroRehabilitation and Neural Repair*, with both Np and Nc ranking among the top five. These journals play a crucial role in advancing the field. The Journal Citation Reports (JCR) quartiles reflect the impact of journals. Journals are categorized into quartiles based on their impact factor, with those in the top 25% (including those at the 25th percentile) classified in JCR Quartile 1 (Q1). Journals ranked between the 25th and 50th percentiles (including those at the 50th percentile) fall into JCR Quartile 2 (Q2). Notably, all 10 journals with the highest publication counts are high-impact journals, classified in Q2 or above, and the co-cited journals are similarly concentrated in high-impact publications, highlighting the significant global academic value of VNS stroke research. By applying Bradford’s Law to the distribution of papers among journals, core journals in the field can be identified. Focusing on these core journals will help improve research efficiency and build a more systematic knowledge base. Furthermore, the cross-disciplinary citation pattern in VNS stroke research suggests that the field not only draws heavily from within its own domain but also incorporates findings from other disciplines, fostering interdisciplinary academic exchange and knowledge integration (Yan and Wang, 2023). Given the interdisciplinary nature of VNS applications, it would be beneficial to encourage collaborations with fields like neurology, bioengineering, and clinical psychology to enhance research scope and innovation.

### 4.2 Research hotspots and frontiers

Bibliometrics plays a crucial role in scientific research by processing and analyzing extensive datasets to offer valuable insights into emerging trends (Chen and Song, 2019). Among its methods, citation analysis is particularly recognized for uncovering research hotspots and trends within distinct fields (Li F. et al., 2023). Through clustering and analyzing the evolutionary trajectories of key research topics in the references (Figures 7C,D), it is evident that current research on VNS in stroke predominantly focuses on two primary areas: exploring the mechanisms of VNS using animal models and investigating its efficacy in improving upper limb sensory function in stroke patients. Beyond identifying references experiencing citation bursts, keyword analysis effectively captures the distribution and development of research hotspots and trends within the VNS-stroke domain. Based on the results from citation burst analysis and keyword timeline mapping, we have categorized the research hotspots into two major areas: VNS mechanism research and clinical application research. Before delving into a detailed analysis, we provide an overview of the distribution of these research hotspots.

1. VNS mechanism research hotspots: This research direction primarily concentrates on exploring VNS mechanisms using animal models. Key keyword clusters include #2 regulatory T cells, #4 rat model, #5 neuroinflammation, and #10 blood–brain barrier. Corresponding reference clusters include #3 cerebral blood flow, #4 spreading depolarizations, #13 blood–brain barrier, #14 nicotinic, #15 neurogenesis, #16 oxidative stress, and #18 sprouting. Collectively, these studies focus on the role of VNS in regulating neural and vascular responses, inflammation, and oxidative stress.
2. VNS clinical application research hotspots: This research direction emphasizes the therapeutic efficacy of VNS in improving upper limb sensory and motor functions in stroke patients. Key keyword clusters include #3 upper limb rehabilitation and #0 plasticity, while reference clusters encompass #0 meta-analysis and #8 non-invasive vagus nerve stimulation. These studies predominantly investigate how VNS enhances neuroplasticity and functional recovery, thereby improving upper limb motor abilities in patients.
3. Potential applications of VNS in post-stroke complications: This research direction explores the potential of VNS to address various post-stroke complications. Key keyword clusters include #6 complications, #7 dysphagia, #8 cognitive function, #9 gastrointestinal dysfunction, #12 depression, and #13 Insomnia. While corresponding reference clusters include #6 microbiota, #9 aphasia, and #11 obstructive sleep. These studies examine the application of VNS in alleviating post-stroke dysphagia, speech disorders, cognitive impairments, post-stroke depression, sleep disturbances, and gut microbiota dysregulation.

#### 4.2.1 Insights into VNS mechanisms for stroke treatment based on animal models

##### 4.2.1.1 VNS inhibits neuroinflammation

VNS has been shown to affect a significant number of vagal neurons, thereby modulating inflammatory responses (Keute and Gharabaghi, 2021). Its impact on central nervous system inflammation is well-documented. For instance, Lu et al. (2017) reported that in a permanent middle cerebral artery occlusion (MCAO) rat model, VNS can stimulate the cholinergic anti-inflammatory pathway, activating T cells to produce acetylcholine (ACh). This ACh subsequently acts on *α*-7 nicotinic acetylcholine receptors (α-7nAChR), predominantly expressed on macrophages, leading to a reduction in pro-inflammatory cytokines such as TNF-α, IL-1β, and IL-6, thereby facilitating the recovery of neurological function following acute brain injury. Moreover, microglia, the resident immune cells of the brain, play a crucial role in post-stroke neurological deficits. Inhibiting their activation has been shown to improve neurological outcomes and reduce brain damage following stroke (Jiang et al., 2020). After cerebral ischemia, ischemic neurons drive microglial polarization toward the pro-inflammatory M1 phenotype, exacerbating ischemic injury (Ma et al., 2017). In contrast, VNS promotes the polarization of microglia toward the M2 phenotype, which not only mitigates ischemic damage but also aids in post-ischemic brain repair by producing anti-inflammatory cytokines such as IL-4 and IL-10, thereby suppressing post-ischemic inflammatory responses (Liu et al., 2023). Furthermore, VNS has been associated with the upregulation of brain-derived neurotrophic factor (BDNF), which further promotes the M2 microglial phenotype (Zhang et al., 2024). Thus, VNS exerts its neuroregulatory effects through multiple pathways, alleviating neuroinflammation within the brain. However, the precise neurobiological mechanisms underlying these effects remain unclear, necessitating further investigation.

##### 4.2.1.2 VNS promotes angiogenesis

The formation of collateral vessels enhances blood supply to peripheral tissues post-focal cerebral ischemia, and angiogenesis is closely linked to neurogenesis, aiding in the recovery of neurological function following ischemic stroke (Sommer, 2017). In animal models of stroke, VNS has been demonstrated to promote angiogenesis, which is vital for improving local cerebral tissue perfusion and fostering neural functional recovery (Capilupi et al., 2020). Li et al. (2020) found that VNS enhances the expression of angiogenic factors such as brain-derived neurotrophic factor (BDNF), endothelial nitric oxide synthase (eNOS), and vascular endothelial growth factor (VEGF), thereby stimulating endothelial cell proliferation, inducing angiogenesis, and increasing microvascular density around the infarcted area. Additionally, another study indicated that VNS stimulates endothelial cell proliferation and increases ALK5 expression in endothelial cells 7 days post-stroke, suggesting that VNS may enhance stroke recovery by increasing the concentration of growth differentiation factor 11 (GDF11) in the vasculature (Ma et al., 2016). These results indicate that VNS promotes angiogenesis through various mechanisms, including upregulating the expression of angiogenic factors and enhancing endothelial cell proliferation, thereby contributing to the recovery of neurological functions post-stroke.

##### 4.2.1.3 VNS modulates blood–brain barrier permeability

The disruption of the Blood–Brain Barrier (BBB) is a pivotal factor in post-stroke neurological dysfunction and is closely associated with poor clinical outcomes during and after a stroke. Yang et al. (2018) demonstrated that, in a MCAO animal model, VNS significantly reduced BBB permeability 24 h post-ischemia/reperfusion, as quantified by dynamic contrast-enhanced magnetic resonance imaging (DCE-MRI). This reduction in permeability contributes to the alleviation of cerebral edema and the preservation of neurological function. Similarly, recent findings suggest that in VNS-treated rats, serum IgG leakage in the ischemic hemisphere is significantly reduced, aligning with immunohistochemistry (IHC) results following MRI (Wang et al., 2024). Furthermore, BBB integrity is maintained by endothelial cells at tight junctions, astrocytic end-feet, pericytes, and the extracellular matrix (Zhuang et al., 2024). Matrix metalloproteinases (MMPs) can degrade tight junction proteins (TJPs), leading to BBB disruption during ischemic stroke. In the ischemic hemisphere, VNS mitigates BBB disruption by reducing the degradation of TJPs such as ZO-1, occludin, and claudin-5 within endothelial cells. Additionally, VNS protects microvascular TJPs by decreasing MMP-2/9 expression in astrocytes surrounding damaged vessels (Rempe et al., 2016). Neren et al. (2016) demonstrated that VNS enhances BBB integrity in rat models of cortical microinfarcts, cortical dysplasia, and traumatic brain injury, underscoring its potential in preserving BBB function post-stroke. In summary, VNS modulates BBB permeability through multiple mechanisms, aiding in the reduction of cerebral edema and the preservation of neurological function, thereby offering a promising therapeutic strategy for stroke management.

##### 4.2.1.4 VNS modulates apoptosis

Apoptosis is a critical factor in post-stroke neuronal death, characterized by DNA fragmentation, degradation of cytoskeletal and nuclear proteins, protein cross-linking, formation of apoptotic bodies, and the expression of phagocytic receptor ligands, ultimately leading to cell clearance by phagocytosis (Tuo et al., 2022). Research by Jiang et al. (2015) demonstrated that VNS significantly reduces the expression of TUNEL, pro-inflammatory cytokines, and cleaved caspase-3, thus playing a pivotal role in mitigating inflammation and apoptosis during ischemic events. Moreover, Zhang et al. (2017) proposed that VNS may further reduce neuronal apoptosis by upregulating prostaglandin D2 synthase in neurons located within the ischemic penumbra. Another significant aspect of apoptosis following stroke is excitotoxicity, which is induced by the excessive accumulation of excitatory neurotransmitters such as glutamate and the overactivation of AMPA receptors on neurons. This process leads to calcium influx and subsequent cellular damage or death. Cheyuo et al. (2011) demonstrated that VNS attenuates excitotoxicity by reducing glutamate release and inhibiting receptor activity, thereby decreasing neuronal damage post-stroke. These findings underscore the therapeutic potential of VNS in promoting post-stroke neural repair. Future research should further investigate the effects of VNS on apoptosis to provide more robust scientific evidence for the development of novel stroke treatments.

##### 4.2.1.5 VNS reduces spreading depolarization

Spreading depolarization (SD) is a pathological event frequently observed following cerebral ischemia, characterized by a sudden and prolonged depolarization of brain tissue that leads to cytotoxic edema, disruption of blood flow, and eventual infarction (Ayata and Lauritzen, 2015). SD is primarily driven by the failure of sodium pumps in ischemic regions, resulting in ion gradient imbalances across cell membranes, abnormal neuronal discharges, and energy depletion (Dreier et al., 2012). Lindemann et al. (2020) demonstrated that VNS significantly reduced the frequency of SD in peri-infarct cortical regions during permanent MCAO without significantly affecting relative blood flow, blood pressure, heart rate, or respiratory rate compared to sham VNS. This suggests that VNS may mitigate the clinical impact of SD waves through direct effects on brain tissue. Although the precise mechanisms by which VNS reduces SD are not yet fully understood, evidence suggests that VNS modulates ion channels and signaling pathways involved in SD by activating the nucleus tractus solitarius (NTS) in the brainstem and influencing neural networks within the brain (Kumaria and Tolias, 2012). Additionally, VNS may indirectly attenuate the effects of SD by enhancing cerebral blood flow, reducing oxidative stress, and dampening inflammatory responses (Wang et al., 2021). These findings underscore the potential of VNS in stroke treatment, suggesting that VNS reduces post-ischemic neuronal injury through a multifaceted approach that diminishes the incidence of SD. However, further research is required to elucidate the specific molecular mechanisms through which VNS influences SD and to evaluate its feasibility and safety in clinical stroke management.

#### 4.2.2 VNS in enhancing post-stroke upper limb sensorimotor function

The integration of VNS with rehabilitation training for the enhancement of upper limb sensorimotor function in stroke patients has garnered significant attention in recent research. Experimental studies involving ischemic and hemorrhagic stroke rat models have demonstrated notable improvements in forelimb strength and movement speed following VNS in combination with rehabilitation therapy (Kimberley et al., 2018, 2019; Dickie et al., 2019; Dawson et al., 2021). These findings provide preliminary evidence supporting the efficacy of VNS in stroke rehabilitation. Furthermore, clinical studies have reinforced these experimental results. For instance, Dawson et al. (2021) reported that stroke patients who underwent iVNS showed superior outcomes in the Fugl-Meyer Assessment for Upper Extremity (FMA-UE) compared to those receiving rehabilitation alone. Additional improvements were also observed in other motor function assessments, such as the Wolf Motor Function Test, the Box and Block Test (BBT), and the Nine-Hole Peg Test (NHPT). With respect to sensory function recovery, regarding the recovery of sensory function, although the majority of existing evidence stems from case reports and preliminary studies, early results appear promising. For instance, Kilgard et al. (2018) documented a case in which VNS combined with tactile therapy resulted in improved sensory function in a male patient with severe sensory deficits in his left arm and hand. The study by Baig et al. (2019) demonstrated that the majority of participants exhibited improvements in light touch and proprioception following the intervention. Furthermore, these enhancements in sensory function were correlated with improvements in motor function. These findings suggest that VNS may promote neuroplastic changes in the brain, thereby contributing to sensory function improvement. The effectiveness of VNS in post-stroke rehabilitation is modulated by several factors, including stimulation intensity, frequency, and duration. Lin et al. (2024) demonstrated that stimulation intensity plays a crucial role in modulating motor cortex plasticity. Similarly, Thompson et al. (2021) emphasized that the therapeutic efficacy of VNS is highly dependent on the specific stimulation parameters employed. Given the variability in patient responses, optimizing stimulation parameters is essential to maximize therapeutic benefits. Typically, stimulation current is set according to the patient’s sensory threshold, with intensities ranging from 0.5 to 6 mA, ensuring it remains below the pain threshold (Wang X. et al., 2023). Gao et al. (2023) indicates that factors such as the stage of stroke, stimulation frequency, duty cycle, pulse count per cycle, frequency of weekly interventions, and the specific combination of VNS parameters are critical in optimizing the therapeutic effects of VNS. Stimulation frequencies between 20 Hz and 30 Hz have been found to be most effective, while adjustments to the stimulation cycle (e.g., 1 min of stimulation followed by 3 min of rest) help mitigate side effects and enhance neural repair. In acute stroke patients, moderate stimulation intensities (typically ranging from 0.25 to 1.0 mA) promote functional recovery with fewer side effects. Furthermore, modulating both stimulation frequency and duration plays a pivotal role in recovery outcomes, with longer stimulation periods (e.g., 10 to 20 min per session) significantly contributing to improved cerebral function. The choice between iVNS and tVNS is primarily guided by the patient’s clinical condition. iVNS involves the surgical implantation of electrodes and a stimulator in the neck for direct VNS. Clinical evidence suggests that iVNS provides neuroprotective effects. However, in acute stroke cases, surgical interventions may be contraindicated, particularly in patients undergoing thrombolysis or anticoagulant therapy. Conversely, tVNS, which involves non-invasive stimulation of the auricular or cervical skin, is more suitable for patients in acute stroke settings, as it does not require surgical procedures. Regarding safety, the profile of VNS has been well-documented. Research conducted by Dawson et al. (2016), Redgrave et al. (2018), and Dawson et al. (2021) indicates that adverse events related to VNS in stroke patients are generally rare. The most common side effects include itching and redness at the stimulation site. Less common side effects include nausea, vomiting, headaches, facial drooping, dizziness, and hoarseness. Although side effects are typically mild, some reports have identified potentially serious complications such as bradycardia and hypotension (Ramos-Castaneda et al., 2022). In conclusion, VNS has emerged as a promising therapeutic modality for stroke rehabilitation. Preliminary evidence supports its efficacy and safety in enhancing upper limb sensory and motor functions post-stroke. However, further research is necessary to optimize treatment protocols, identify ideal stimulation parameters and timing, and address individual patient variability to facilitate broader clinical adoption.

#### 4.2.3 Beyond motor functions of VNS in stroke rehabilitation

##### 4.2.3.1 Management of dysphagia and speech disorders

Dysphagia is a prevalent post-stroke complication, affecting between 37 and 78% of patients. This condition significantly elevates the risk of pneumonia, dehydration, and malnutrition, thereby prolonging hospital stays and escalating healthcare costs (Cohen et al., 2016). Animal studies indicate that VNS can substantially enhance oral feeding capacity and reduce residual saliva in patients with post-stroke dysphagia (Long et al., 2022). Yuan et al. (2019) conducted a study involving a case of severe dysphagia following dorsal medullary infarction, where the patient received percutaneous VNS therapy for six weeks. Post-treatment, the patient exhibited remarkable improvement, achieving the ability to consume a full oral diet. This finding provides preliminary evidence supporting the potential efficacy of taVNS in treating severe dysphagia resulting from vagal nuclear damage. Additional evidence is provided by a preliminary study conducted by Wang Y. et al. (2023), which reported that the active taVNS treatment group displayed significantly greater improvements on the swallowing function assessment scale compared to the control group, with these improvements persisting for at least 4 weeks post-treatment. No serious adverse events were observed during the study, suggesting that taVNS is a safe and well-tolerated therapeutic approach. Despite these encouraging results, the current clinical evidence remains limited. Therefore, further research is necessary to validate the efficacy and safety of VNS in the treatment of post-stroke dysphagia. Given the considerable overlap in the neural control centers for motor speech and swallowing, as well as their proximity to the brain regions governing limb movement, VNS may also serve as an adjunctive training method to enhance speech function (Wang et al., 2020). Recent studies have demonstrated that VNS can improve the functionality of muscle and nerve circuits in the brain, enhance plasticity within the cortical circuits responsible for upper limb movement, and promote synaptic plasticity involved in regulating lip movements (Morrison et al., 2021).

##### 4.2.3.2 Cognitive function restoration

The restoration of cognitive function is pivotal for stroke survivors to achieve independence in daily activities. VNS has demonstrated potential in enhancing cognitive function by targeting brain regions intimately associated with cognition, such as the thalamus, hippocampus, and amygdala (Li K.-P. et al., 2023). In a study conducted by Colombo et al. (2023), the impact of transcutaneous tVNS on cognitive function in stroke patients was evaluated using the Go/No-go test, which measures motor preparedness and response inhibition—two essential aspects of cognitive function. The results indicated a positive trend in cognitive function improvement following a single tVNS session compared to the sham stimulation condition. However, the mean latency of Go/No-go tests between the two groups did not exhibit statistically significant differences. Liu W. et al. (2024) preliminarily explored the combined application of tDCS and taVNS for its efficacy and underlying neural mechanisms in post-stroke cognitive impairment patients, utilizing the Montreal Cognitive Assessment and functional magnetic resonance imaging. Additionally, animal experiments conducted by Wu et al. (2018) revealed that tVNS could enhance cognitive function in post-stroke rats, potentially through modulating acetylcholine release and uptake in the non-neuronal cholinergic system (NNCS). In these experiments, tVNS reversed the neural damage induced by middle cerebral artery occlusion and increased the expression of cholinergic-related enzymes and transport proteins. These findings suggest that VNS may enhance cognitive function by promoting brain tissue protection and repair; however, its precise neurobiological mechanisms remain to be elucidated. Future research should focus on the long-term effects of VNS on cognitive function, optimizing stimulation parameters to maximize therapeutic outcomes, and conducting rigorously designed clinical trials to evaluate the safety and efficacy of VNS across diverse stroke patient populations.

##### 4.2.3.3 Addressing post-stroke insomnia and psychiatric disorders

Insomnia affects approximately 37–59% of stroke patients and is associated with an increased incidence of cardiovascular diseases and diminished stroke recovery outcomes (Cao et al., 2024). Zhao et al. (2019) were the first to explore the efficacy of taVNS in treating post-stroke insomnia (PSI). Following taVNS treatment, patients exhibited significant improvements in sleep quality and duration. Functional MRI revealed changes in the functional connectivity of brain networks involved in sleep regulation. This study posits that taVNS is a safe and effective therapeutic option for PSI and advocates for broader clinical trials to validate these findings. Future research should focus on optimizing taVNS protocols and stimulation parameters, as well as confirming its long-term efficacy in patients with post-stroke insomnia. Common post-stroke psychiatric disorders, such as anxiety, depression, and substance use disorders, are potentially linked to maladaptive plasticity in brain nociceptors. Neuroimaging evidence often shows weakened functional connectivity between the basal ganglia and frontal-thalamic circuits (Shi et al., 2019). taVNS treatment can enhance connectivity in emotion-related brain regions, such as between the posterior cingulate cortex and basal ganglia, providing a novel, portable, self-administered, and safe treatment option for patients with post-stroke psychiatric disorders (Cheng et al., 2022), investigated the safety, efficacy, and molecular mechanisms of taVNS in treating post-stroke depression (PSD). Results indicated that the taVNS group showed significant improvements in scores on the Hamilton Depression Rating Scale (HAMD-17), Zung Self-Rating Depression Scale (SDS), and Barthel Index (BI), with enhanced daily living functions. The taVNS group also demonstrated superior outcomes in regulating neurotrophic biomarkers compared to the control group. Moreover, VNS has shown promising efficacy in treating treatment-resistant depression Rudas et al. (2012), suggesting that, when combined with appropriate behavioral or cognitive therapies, VNS may effectively improve post-stroke psychiatric and sleep disorders.

##### 4.2.3.4 Enhancing gastrointestinal dysfunction

More than 50% of ischemic stroke survivors encounter gastrointestinal complications, including dysbiosis, constipation, and gastrointestinal bleeding. These conditions exacerbate post-stroke adverse outcomes through the bidirectional transmission of pro-inflammatory cells and metabolites between the gut and the brain, ultimately leading to gut-brain axis dysfunction (Yong et al., 2023). The vagus nerve-brain-immune axis (VNBIA) constitutes a complex physiological network that plays a critical role in managing gastrointestinal dysfunction following a stroke (van der Meij and Wermer, 2021). Recent research has demonstrated that VNS can mitigate gastrointestinal dysbiosis and reduce both intestinal and neuroinflammation by inhibiting mast cell (MC) degranulation and decreasing trypsin secretion. This modulation enhances the integrity of the gut and BBB, alleviates systemic inflammatory responses, and improves gastrointestinal function post-stroke (Wang et al., 2024). Furthermore, studies by Benakis and Liesz (2022) indicate that VNS positively affects the gut microbiota, aiding in the restoration of gut microecological balance and reducing intestinal inflammation. Clinically, VNS presents a promising therapeutic strategy for managing gastrointestinal dysfunction in post-stroke patients. Future research should focus on optimizing VNS protocols and confirming its long-term efficacy across diverse patient populations.

#### 4.2.4 Limitations in clinical applications

Although VNS holds significant promise in stroke rehabilitation, its application is constrained by various limitations and challenges. These include cost management, improving accessibility, addressing potential side effects, developing individualized treatment strategies, and managing complex complications effectively. From an economic perspective, implantable iVNS imposes a substantial financial burden on both patients and healthcare systems due to the high costs of surgical implantation and monitoring equipment. While tVNS offers a more cost-effective alternative initially, the cumulative expenses over prolonged usage cannot be overlooked. Regarding accessibility and suitability, the adoption of VNS is hindered by disparities in medical resources and the uneven dissemination of advanced technology, particularly in resource-limited regions. For acute stroke patients undergoing thrombolysis or anticoagulation therapy, iVNS may be contraindicated due to the heightened risk of bleeding, making tVNS a preferable option in such circumstances. Despite its generally favorable safety profile, VNS is not without side effects. Common adverse reactions, such as localized itching, erythema at the stimulation site, and occasional instances of nausea, vomiting, headache, or facial droop, warrant careful monitoring. Moreover, the potential long-term impact of VNS on cardiovascular health, such as alterations in blood pressure and heart rate, necessitates ongoing surveillance (Korupolu et al., 2024). The importance of individualized treatment cannot be overstated. Tailoring VNS protocols requires consideration of patient-specific factors such as age, sex, and the type and location of the stroke. Additionally, further investigation into the underlying mechanisms of VNS on neural networks is essential to refine therapeutic strategies and enhance precision. Finally, while VNS shows potential in addressing post-stroke complications such as dysphagia, cognitive dysfunction, and depressive symptoms, the effective management of these complex issues demands a multidisciplinary approach. The presence of comorbid conditions may also modulate the efficacy of VNS interventions. Future research should prioritize the development of optimized treatment protocols, cost-effective VNS devices, and a deeper understanding of the neurobiological mechanisms underlying its effects. These efforts are crucial for advancing the clinical application of VNS in stroke rehabilitation and providing patients with more diverse and accessible therapeutic options.

### 4.3 Limitations

This study has several limitations. Firstly, all data were sourced from the WoSCC, excluding other databases such as PubMed and Embase. Although WoSCC includes most significant publications, some unindexed literature may have been missed, potentially affecting the comprehensiveness and representativeness of our analysis results. Secondly, this study only included English-language papers and reviews, and there were quality variations among the selected literature, which may somewhat undermine the reliability of the analysis results. Additionally, bibliometric analysis tools possess inherent limitations. For instance, during clustering analysis, terms extracted based on article titles, abstracts, and keywords can exhibit significant variability, and the integration of synonyms is not always entirely accurate, which might affect the precision of the analysis results.

## 5 Conclusion

Globally, the application of VNS in stroke research has been advancing steadily, with significant contributions from the USA, China, and several European countries. The journal *Frontiers in Neuroscience* has emerged as a pivotal academic platform in this domain. Professor Michael P. Kilgard from the University of Texas has made considerable strides in investigating the role of VNS in stroke rehabilitation. Currently, the neuroprotective effects of VNS in stroke treatment, including its ability to inhibit neuroinflammation, protect the blood–brain barrier, and promote angiogenesis, have become central themes in foundational research. Given the physiological and pathological similarities between rats and humans, the rat model has been widely employed in these studies. Future efforts should focus on optimizing these models through genetic editing techniques to explore the roles of specific genes in mediating the effects of VNS. Non-human primate models, due to their close anatomical and physiological resemblance to humans, may further enhance the clinical relevance of VNS research. The therapeutic efficacy of VNS is closely linked to the stimulation parameters. Future investigations should prioritize the development of individualized stimulation protocols and the exploration of novel stimulation patterns, such as frequency modulation, to improve treatment outcomes. Long-term follow-up studies and the management of stroke-related complications, including dysphagia, speech and cognitive impairments, insomnia, depression, and gut microbiota dysregulation, are currently focal points of ongoing research. However, the clinical application of VNS faces several challenges, including technical complexity, clinical translation, long-term efficacy, and patient adherence. Adverse reactions and safety concerns also remain critical areas of investigation. Future research should focus on optimizing VNS parameters, developing new models, evaluating post-stroke outcomes, and enhancing both clinical application and safety to provide more effective treatment options for stroke patients. Overall, this bibliometric analysis offers a significant objective perspective, enabling researchers to better track cutting-edge knowledge and emerging trends in VNS-related stroke therapy.
