# Supplementary material for: Research hotspots and frontiers of vagus nerve stimulation in stroke: a bibliometric analysis
Source: Front Neurosci. 2024 Dec 11;18:1510658. doi: 10.3389/fnins.2024.1510658 (PMC11668697; doi:10.3389/fnins.2024.1510658)
Supplement: Supplementary file 1 [file Data_Sheet_1.PDF]

Supplementary Material

1 Search Strategy

Search Date: September 1, 2024, 13:30

Search Time Range: January 1, 2005, to August 31, 2024

Search Language: English

#1 ((((((((TS=(Vagus Nerve Stimulation*)) OR TS=(Stimulation*, Vagus Nerve)) OR TS=(Nerve Stimulation*, Vagus)) OR TS=(Vagal Nerve Stimulation*)) OR TS=(Stimulation*, Vagal Nerve)) OR TS=(Nerve Stimulation*, Vagal)) OR TS=(Transcutaneous auricular vagus nerve stimulation*)) OR TS=(Transcutaneous vagus nerve stimulation*)) OR TS=(Noninvasive vagus nerve stimulation*)) OR TS=(Transcutaneous cervical vagus nerve stimulation*))

#2 ((((((((((((TS=(Stroke*)) OR TS=(Cerebrovascular Accident*)) OR TS=(Cerebral Stroke*)) OR TS=(Cerebrovascular Apoplexy)) OR TS=(Brain Vascular Accident*)) OR TS=(Cerebrovascular Stroke*)) OR TS=(Apoplexy)) OR TS=(Acute Stroke*)) OR TS=(Acute Cerebrovascular Accident*)) OR TS=(Ischemic Stroke*)) OR TS=(Brain Stem Infarction)) OR TS=(Hemorrhagic Stroke*)) OR TS=(Intracerebral Hemorrhagic Stroke*)) OR TS=(Subarachnoid Hemorrhagic Stroke*))

#3 #1 AND #2

2 Data Preprocessing Procedures

2.1 Institutional Integration:

University of Texas System

Merged Entities: University of Texas at Dallas, University of Texas Health Science Center at Houston, and University of Texas Southwestern Medical Center at Dallas—all distinct institutions under the University of Texas System.

University of California System

Merged Entities: University of California, Los Angeles; University of California, Irvine; and University of California, San Francisco—universities within the University of California System.

University of Minnesota System

Merged Entities: University of Minnesota, Twin Cities—different campuses unified under the University of Minnesota System.

Chinese Academy of Sciences

Merged Entities: University of Chinese Academy of Sciences and China Academy of Chinese Medical Sciences—both affiliated with the Chinese Academy of Sciences.

Veterans Health Administration (VHA)

Merged Entities: U.S. Department of Veterans Affairs—components of the broader healthcare system managed by the Department of Veterans Affairs.

Harvard University

Merged Entities: Harvard Medical School and Massachusetts General Hospital—affiliated entities within Harvard University.

Weill Cornell Medicine

Merged Entities: Cornell University—encompassing the university and its medical school.

University of Wisconsin System

Merged Entities: University of Wisconsin, Madison—different campuses consolidated under the University of Wisconsin System.

2.2 Country Classification:

Americas: United States (USA), Canada, Mexico, Brazil, Colombia, and Chile.

Europe: England, Scotland, Germany, Italy, France, Belgium, Netherlands, Turkey, Switzerland, Sweden, Denmark, Czech Republic, Austria, Ireland, Russia, Slovakia, Slovenia, Luxembourg, Romania, Spain, Portugal, Finland, Poland, and Hungary.

Asia: People's Republic of China, Japan, South Korea, India, Iran, and Saudi Arabia.

2.3 Author Confirmation:

We have verified the following authors through the article abstracts:

Bashar W. Badran: Badran, Bashar W.

Changqing Li: Li, Changqing

Seth Hays: Hays, Seth A.

Robert L. Rennaker: Rennaker, Robert L. and Rennaker II, Robert L.

Teresa J. Kimberley: Kimberley, Teresa J. and Kimberley, Teresa Jacobson

Navzer Engineer: Engineer, Navzer D. and Engineer, Navzer.

2.4 Consolidation of Keywords

Vagus Nerve Stimulation (VNS) : vagus nerve stimulation, vagal nerve stimulation, vagal stimulation, VNS

3 Graphical Interpretation

Node Types: In CiteSpace, nodes represent distinct elements in bibliometric analysis, such as authors, institutions, countries, keywords, and documents. Different node types are distinguished by various shapes and colors, facilitating their identification.

Node Size: The size of a node typically correlates with a specific metric associated with that node, such as the number of publications or citation frequency. Larger nodes indicate greater importance or influence of that node within the network.

Links: Links denote the relationships between nodes, such as co-occurrence or citation connections. The thickness and color of the links generally represent the strength or frequency of these relationships. A thicker link indicates a stronger relationship or higher co-occurrence frequency, while a thinner link denotes a weaker relationship or lower frequency.

Color Coding: Color coding is commonly used to represent time or specific attributes. In terms of the temporal dimension, a color gradient from cool tones (e.g., blue) representing earlier periods to warm tones (e.g., red) representing more recent periods illustrates the temporal evolution of nodes or links. For cluster analysis, different colors signify different clusters or research themes.

Cluster Analysis: CiteSpace employs cluster analysis to group related documents or keywords into distinct themes or research areas. Each cluster is labeled, typically based on the keywords or subject terms within the documents of that cluster. The boundaries of these clusters are usually marked by color or shape to facilitate differentiation.

Burst Terms: Burst terms refer to words or phrases that experience a sharp increase in frequency over a specific time period, often reflecting emerging research trends or hot topics in a field. A red outer ring denotes the burst status of a node within a particular timeframe, with the thickness of the ring indicating the degree of burstiness.

Betweenness Centrality: Betweenness centrality is a key network analysis metric in CiteSpace, used to measure the significance of a node in the network, particularly its role in connecting the shortest paths between other nodes. The concept of betweenness centrality, introduced by sociologist Linton Freeman, quantifies the extent to which a node acts as a "broker" in the network. Nodes that lie on the shortest paths between multiple node pairs possess higher betweenness centrality, signifying their crucial role in connecting disparate points within the network. In CiteSpace, nodes with a betweenness centrality greater than 0.1 are considered key nodes, serving as bridges that link different perspectives, themes, or research areas. Nodes with high betweenness centrality are marked with a purple ring, with the thickness of the ring corresponding to the magnitude of the centrality value. Such nodes are typically positioned at the intersection of two large clusters or sub-networks, playing a pivotal "mediator" role.

Timeline View: The timeline view illustrates the evolution of documents or keywords over time, aiding in the identification of changing research trends. The horizontal axis represents time, while the vertical axis indicates documents, keywords, or themes.

Knowledge Flow: Knowledge flow diagrams depict the movement of knowledge from one field or theme to another. Arrows or gradient lines indicate the pathways of knowledge dissemination, providing insights into the exchange and interaction of knowledge across different domains.

Dual Map Overlay: The dual map overlay consists of two foundational maps. The left map typically represents the distribution of cited documents (i.e., documents that have been cited), while the right map shows the distribution of reference documents (i.e., documents that are cited). The overlay map displays the citation relationships between fields (or journals), with each map divided into regions based on the subject classification of the journals or the thematic clustering of the documents. The size of each region reflects the volume or impact of literature within that field or journal, while the color may denote different subject categories or clusters. Connecting lines or curves represent citation relationships, with thicker lines indicating stronger citation relationships and thinner lines indicating weaker ones. Colors often correspond to the temporal dimension or other properties of the citation relationships. Through the overlay effect, researchers can visually discern citation patterns between different fields or journals, thereby uncovering the flow and direction of knowledge.
